# Molecular Heterogeneity in Localized Diffuse Large B-Cell Lymphoma

**DOI:** 10.3389/fonc.2021.638757

**Published:** 2021-09-07

**Authors:** Wei Qin, Di Fu, Qing Shi, Lei Dong, Hongmei Yi, Hengye Huang, Xufeng Jiang, Qi Song, Zhenhua Liu, Shu Cheng, Jinyan Huang, Li Wang, Pengpeng Xu, Weili Zhao

**Affiliations:** ^1^Shanghai Institute of Hematology, State Key Laboratory of Medical Genomics, National Research Center for Translational Medicine, Rui Jin Hospital Affiliated to Shanghai Jiao Tong University School of Medicine, Shanghai, China; ^2^Department of Pathology, Shanghai Rui Jin Hospital, Shanghai Jiao Tong University School of Medicine, Shanghai, China; ^3^School of Public Health, Shanghai Jiao Tong University School of Medicine, Shanghai, China; ^4^Department of Nuclear Medicine, Shanghai Rui Jin Hospital, Shanghai Jiao Tong University School of Medicine, Shanghai, China; ^5^Department of Radiology, Shanghai Rui Jin Hospital, Shanghai Jiao Tong University School of Medicine, Shanghai, China; ^6^Department of Ultrasound, Shanghai Rui Jin Hospital, Shanghai Jiao Tong University School of Medicine, Shanghai, China; ^7^Laboratory of Molecular Pathology, Pôle de Recherches Sino-Français en Science du Vivant et Génomique, Shanghai, China

**Keywords:** diffuse large B-cell lymphoma, single nodal, single extranodal, serum lactate dehydrogenase, gene mutations, tumor microenvironment

## Abstract

The clinical and molecular characteristics of localized diffuse large B-cell lymphoma (DLBCL) with single nodal (SN) or single extranodal (SE) involvement remain largely elusive in the rituximab era. The clinical data of 181 patients from a retrospective cohort and 108 patients from a phase 3 randomized trial NHL-001 (NCT01852435) were reviewed. Meanwhile, genetic aberrations, gene expression pattern, and tumor immunophenotype profile were revealed by DNA and RNA sequencing of 116 and 53 patients, respectively. SE patients showed similar clinicopathological features as SN patients, except for an increased percentage of low-intermediate risk in the National Comprehensive Cancer Network–International Prognostic Index. According to the molecular features, increased *MPEG1* mutations were observed in SN patients, while SE patients were associated with upregulation of TGF-β signaling pathway and downregulation of T-cell receptor signaling pathway. SE patients also presented immunosuppressive status with lower activity of killing of cancer cells and recruiting dendritic cells. Extranodal involvement had no influence on progression-free survival (PFS) or overall survival (OS) in localized DLBCL. Serum lactate dehydrogenase >3 upper limit of normal was an independent adverse prognostic factor for OS, and *ATM* mutations were related to inferior PFS. Although the overall prognosis is satisfactory, specific clinical, genetic, and microenvironmental factors should be considered for future personalized treatment in localized DLBCL.

## Introduction

Diffuse large B-cell lymphoma (DLBCL) is the most common subtype of non-Hodgkin's lymphoma and represents a heterogeneous entity with various clinical, immunophenotypic, and molecular features (1, 2). Anti-CD20 monoclonal antibody rituximab in combination with cyclophosphamide, doxorubicin, vincristine, and prednisone (R-CHOP) has significantly improved the outcome of DLBCL patients (3), particularly in the low-risk group of International Prognostic Index (IPI). In addition to IPI (4), National Comprehensive Cancer Network (NCCN)-IPI has recently been established, stratifying patients according to more refined age range and serum lactate dehydrogenase (LDH) level as well as specific exranodal sites including the gastrointestinal (GI) tract, central nervous system (CNS), liver, lung, and bone marrow (5). In a pathological setting, cell of origin (COO) subtype as germinal center B-cell (GCB) and non-GCB (6), as well as BCL2 (≥50%) and MYC (≥40%) double expressors (7), are recognized as important prognostic factors in DLBCL. However, the clinical characteristics and prognostic features of localized DLBCL remain largely elusive in the rituximab era since these patients respond well to R-CHOP immunochemotherapy and are often excluded from clinical trials of DLBCL.

According to involved sites, localized DLBCL is divided into single nodal (SN) and single extranodal (SE) group. Other than lymph node, Waldeyer's ring and spleen are considered as nodal tissue (8), while GI tract, breast, and CNS are the most common extranodal sites. More recently, extranodal involvement has been identified as an important prognostic factor for inferior survival in localized DLBCL (9), suggesting the potential heterogeneity between nodal and extranodal involvement. Distinct gene mutations have been related to specific extranodal sites of DLBCL. For example, mutations in *MYD88* and *CD79B* were frequently observed in primary CNS, breast, female genital tract, and testicular DLBCL (10, 11) but rarely in primary GI tract DLBCL (12, 13). In addition to lymphoma cells themselves, the tumor microenvironment is essential for tumorigenesis and tumor progression in DLBCL (14). Therefore, the genetic and microenvironmental heterogeneity of localized DLBCL needs to be further investigated.

In the present study, we analyzed the clinical characteristics and prognostic features of localized DLBCL both in retrospective and prospective cohorts, and evaluated the molecular heterogeneity between SN and SE including genetic aberrations, gene expression pattern, and tumor microenvironment profile, which may be helpful for future personalized treatment in localized DLBCL.

## Patients and Methods

### Patients

**Figure 1:**
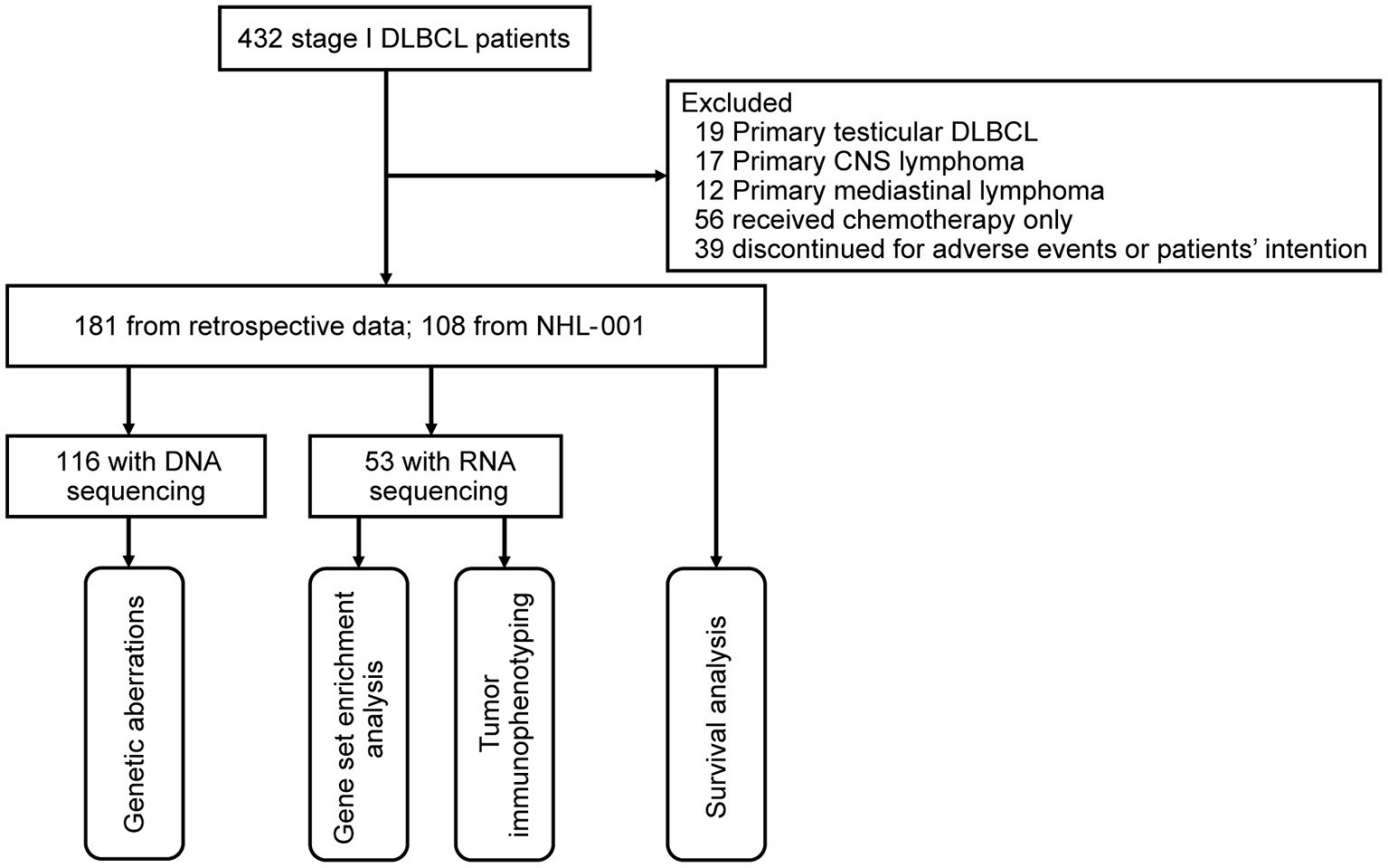
Flow chart describing the cohort selection. DLBCL, diffuse large B-cell lymphoma; CNS, central nervous system.

From April 2003 to February 2019, a total of 432 stage I patients with newly diagnosed DLBCL were included in this study. Histological diagnoses were reviewed according to the World Health Organization 2016 classification (15). A flow chart describing the cohort selection is outlined in Figure 1. Excluding 19 patients with primary testicular DLBCL, 17 patients with primary CNS lymphoma, 12 patients with primary mediastinal B-cell lymphoma, 56 patients receiving chemotherapy alone, and 39 patients who discontinued treatment for adverse events or patients' intention, a total of 289 patients receiving R-CHOP regimen were analyzed. Among them, 181 patients were retrospectively reviewed, and 108 patients were from a prospective phase 3 trial NHL-001 (NCT01852435) randomly receiving R-CHOP50 (doxorubicin 50 mg/m^2^), R-CEOP70 (epirubicin 70 mg/m^2^), or R-CEOP90 (epirubicin 90 mg/m^2^) regimen as previously described (16). DNA sequencing was performed on 116 patients for detection of genetic aberrations, and RNA sequencing was carried out on 53 patients for gene set enrichment analysis and tumor immunophenotyping (TIP). The study was approved by the Ruijin Hospital Ethics Committee, with written informed consent obtained in accordance with the Declaration of Helsinki.

### Clinical and Pathological Data Collections

The following clinical data were collected: age, Eastern Cooperative Oncology Group (ECOG) performance status, serum LDH, IPI, NCCN-IPI, and bulky tumors (>7.5 cm). A total of 14 common sites with lymphoma involvement were analyzed, including lymph node, Waldeyer's ring, spleen, GI tract, breast, skin, bone, thyroid, ovary, nasal, lung, salivary glands, liver, and adrenal, as previously described (17). Immunohistochemistry was performed on 5 μm paraffin sections with an indirect immunoperoxidase method using antibodies against CD10, BCL6, MUM1, BCL2, and MYC. GCB or non-GCB origin was determined using Hans algorithm (6), with 30% cutoff values of CD10, BCL6, and MUM1. As for BCL2/MYC double expressors, the cutoff values of BCL2 and MYC were 50 and 40%, respectively, as previously reported (15).

### DNA and RNA Sequencing

For frozen tumor tissue samples, genomic DNA was extracted using a QIAamp DNA Mini Kit (Qiagen, Hilden, Germany). For formalin-fixed paraffin-embedded (FFPE) samples, genomic DNA was extracted using a GeneRead DNA FFPE Tissue Kit (Qiagen). Targeted sequencing (*n* = 51), whole-exome sequencing (WES) (*n* = 51), or whole-genome sequencing (WGS) (*n* = 14) was performed on 116 patients (including 52 SN and 64 SE patients) with frozen or FFPE tumor tissue samples. Among 65 patients with WES or WGS, the DNA sequencing data of 64 patients were from our previous report on extranodal DLBCL (17), and the data of one patient were newly added. For WGS, the library was validated by Agilent 2100 Bioanalyzer, and sequencing was performed on Illumina HiSeq platform with 150-bp paired-end strategy in WuXi NextCODE, Shanghai. For WES, the exome regions were captured by a SeqCap EZ Human Exome kit (version 3.0), and sequencing was performed on HiSeq 4000 platform with 150-bp paired-end strategy in Righton, Shanghai. As for targeted sequencing, PCR primers were designed by Primer 5.0 software. Multiplexed libraries of tagged amplicons from tumor tissue samples were generated by Shanghai Righton Bio-Pharmaceutical Multiplex-PCR Amplification System. GATK Haplotype Caller and GATK Unified Genotyper were applied to call single nucleotide variations (SNVs) and indels. SNVs reported with low confidence defined by depth (<10) and variant allele frequency (<0.05) were excluded. WGS (*n* = 17) and WES (*n* = 25) were performed on 42 matched peripheral blood samples to exclude germ-line polymorphisms. The detailed procedures for DNA sequencing and variant calling were carried out as previously described (17).

RNA was extracted with Trizol and RNeasy Mini kit (Qiagen) using frozen tumor tissue samples. RNA sequencing was performed on 53 patients (including 32 SN and 21 SE patients). Among them, the RNA sequencing data of 47 patients were from our previous report on extranodal DLBCL (17), and the data of six patients were newly added. RNA purification, reverse transcription, library construction, and sequencing were performed in WuXi NextCODE according to the manufacturer's instructions (Illumina). The detailed procedures for RNA sequencing were conducted as previously described (17). Gene enrichment analysis was performed by overlapping the genes in a module with Kyoto Encyclopedia of Genes and Genomes gene sets using GSEA (v4.0.3) with the C2 collection of the MsigDB (18, 19). A web server TIP was applied to evaluate tumor microenvironment using RNA sequencing data (20).

### Statistical Analysis

The baseline and molecular characteristics of patients were analyzed using Pearson's χ^2^-test or Fisher's exact-test for qualitative data and independent-sample *t*-test or Mann–Whitney *U*-test for quantitative data. Progression-free survival (PFS) was calculated from the date of diagnosis to the date when the disease progression was recognized or the date of last follow-up (March 1, 2020). Overall survival (OS) was measured from the date of diagnosis to the date of death or the date of last follow-up. Survival analyses were estimated using the Kaplan–Meier method and compared by log-rank test. Univariate hazard estimates were generated with unadjusted Cox proportional hazards models. Clinical and pathological covariates demonstrating significance with *P*-value < 0.100 on univariate analysis were included in the multivariate model. Statistical significance was defined as *P*-value < 0.050. All statistical analyses were carried out using *R* software (version 3.6.1; http://www.R-project.org) and Statistical Package for the Social Sciences (SPSS) 22.0 software (SPSS Inc., Chicago, USA).

## Results

### Clinical and Pathological Characteristics

As listed in Table 1, 56.4% of localized DLBCL patients had the origin coming from extranodal sites. GI tract and lymph node were the common sites of involvement, with percentages of 37.0 and 32.9%, respectively. The main characteristics of localized DLBCL patients are summarized in Table 2. Clinically, most patients were featured with a young age (≤60), good ECOG performance status, no bulky tumors, normal serum LDH level, and low-risk IPI and NCCN-IPI. Pathologically, the GCB origin of 44.9% and the BCL2/MYC double expressors of 14.5% were observed. More SE patients were categorized as low-intermediate risk NCCN-IPI than SN patients, both in the retrospective cohort (72.5 vs. 43.1%, *P* < 0.001) and in the prospective cohort (61.1 vs. 37.0%, *P* = 0.044).

**Table 1:** Distribution of specific sites of involvement in patients with localized DLBCL (*n* = 289).

**Site of involvement (*n* = 289)**	**Number (%) of patients**
**SN**	**126 (43.6)**
Lymph node	95 (32.9)
Waldeyer's ring	29 (10.0)
Spleen	2 (0.7)
**SE**	**163 (56.4)**
GI tract	107 (37.0)
Breast	24 (8.3)
Skin	7 (2.4)
Bone	6 (2.1)
Thyroid	4 (1.4)
Ovary	4 (1.4)
Nasal	4 (1.4)
Lung	3 (1.0)
Salivary glands	2 (0.7)
Liver	1 (0.3)
Adrenal	1 (0.3)

*DLBCL, diffuse large B-cell lymphoma; SN, single nodal; SE, single extranodal; GI tract, gastrointestinal tract*.

**Table 2:** Clinical and pathological characteristics of patients with localized DLBCL (*n* = 289).

	**Number (%) of patients**	**Retrospective (** ***n*** **=** **181)**			**NHL-001 (** ***n*** **=** **108)**		
		**SN (*n* = 72)** ***n* (%)**	**SE (*n* = 109)** ***n* (%)**	***P*-value^a^**	**SN (*n* = 54)** ***n* (%)**	**SE (*n* = 54)** ***n* (%)**	***P*-value^b^**
**Age**							
≤ 40 years	52 (18.0)	12 (16.7)	18 (16.5)	0.930	10 (18.5)	12 (22.2)	0.540
41–60 years	138 (47.8)	33 (45.8)	54 (49.5)		23 (42.6)	28 (51.9)	
61–75 years	91 (31.5)	24 (33.3)	34 (31.2)		20 (37.0)	13 (24.1)	
>75 years	8 (2.8)	3 (4.2)	3 (2.8)		1 (1.9)	1 (1.9)	
**Performance status**							
ECOG 0–1	281 (97.2)	70 (97.2)	109 (100.0)	0.157	53 (98.1)	49 (90.7)	0.208
ECOG >1	8 (2.8)	2 (2.8)	0 (0.0)		1 (1.9)	5 (9.3)	
**Serum LDH**							
≤ULN	239 (82.7)	58 (80.6)	87 (79.8)	0.940	46 (85.2)	48 (88.9)	0.209
>1 to ≤3 ULN	45 (15.6)	13 (18.1)	21 (19.3)		5 (9.3)	6 (11.1)	
>3 ULN	5 (1.7)	1 (1.4)	1 (0.9)		3 (5.6)	0 (0.0)	
**Specific extranodal sites** ^**c**^							
No	178 (61.6)	72 (100.0)	35 (32.1)	<0.001	54 (100.0)	17 (31.5)	<0.001
Yes	111 (38.4)	0 (0.0)	74 (67.9)		0 (0.0)	37 (68.5)	
**IPI**							
Low risk	271 (93.8)	65 (90.3)	106 (97.2)	0.094	48 (88.9)	52 (96.3)	0.270
Low-intermediate risk	18 (6.2)	7 (9.7)	3 (2.8)		6 (11.1)	2 (3.7)	
**NCCN-IPI**							
Low risk	114 (39.4)	38 (52.8)	26 (23.9)	<0.001	31 (57.4)	19 (35.2)	0.044
Low-intermediate risk	163 (56.4)	31 (43.1)	79 (72.5)		20 (37.0)	33 (61.1)	
Intermediate-high risk	12 (4.2)	3 (4.2)	4 (3.7)		3 (5.6)	2 (3.7)	
**Bulky tumors**							
No	278 (96.2)	70 (97.2)	106 (97.2)	1.000	50 (92.6)	52 (96.3)	0.674
Yes	11 (3.8)	2 (2.8)	3 (2.8)		4 (7.4)	2 (3.7)	
**Hans (** ***n*** **=** **256)**							
GCB	115 (44.9)	24 (39.3)	45 (48.9)	0.244	26 (51.0)	20 (38.5)	0.201
Non-GCB	141 (55.1)	37 (60.7)	47 (51.1)		25 (49.0)	32 (61.5)	
**DE (** ***n*** **=** **249)**							
No	213 (85.5)	61 (92.4)	91 (91.0)	0.747	30 (73.2)	31 (73.8)	0.947
Yes	36 (14.5)	5 (7.6)	9 (9.0)		11 (26.8)	11 (26.2)	

a *P-value indicated the differences between SN and SE in the retrospective cohort*.

b *P-value indicated the differences between SN and SE in the NHL-001 cohort*.

c *Extranodal sites of gastrointestinal tract, liver, and lung according to NCCN-IPI*.

*DLBCL, diffuse large B-cell lymphoma; SN, single nodal; SE, single extranodal; ECOG, Eastern Cooperative Oncology Group; LDH, lactate dehydrogenase; ULN, upper limit of normal; IPI, International Prognostic Index; NCCN-IPI, National Comprehensive Cancer Network-International Prognostic Index; GCB, germinal center B-cell; DE, BCL2/MYC double expressors*.

### Molecular Characteristics

#### Genetic Aberrations

**Figure 2:**
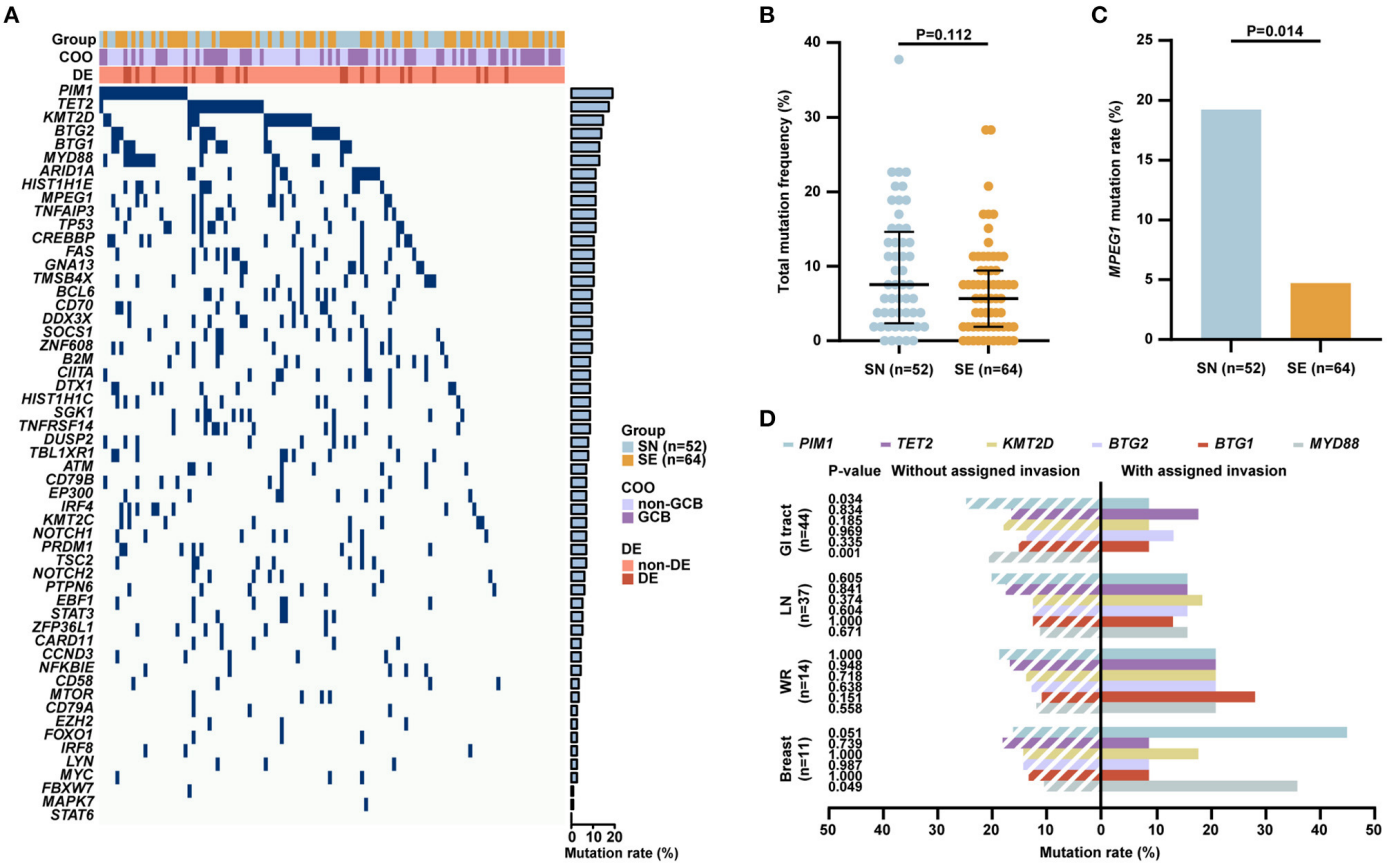
Genetic aberrations of patients with localized DLBCL (*n* = 116). **(A)** Gene mutations in patients with localized DLBCL identified by next-generation sequencing, including 52 patients with SN and 64 patients with SE involvement. **(B)** Total mutation frequency of SN and SE patients. **(C)** Mutation rates of *MPEG1* in SN and SE patients. **(D)** Mutation rates of *PIM1, TET2, KMT2D, BTG2, BTG1*, and *MYD88* in common sites of involvement including GI tract (*n* = 44), lymph node (*n* = 37), Waldeyer's ring (*n* = 14), and breast (*n* = 11). Error bars represent lower and upper quartiles. *P*-values were calculated using Pearson's χ^2^-test or Fisher's exact-test for qualitative data and Mann–Whitney *U*-test for quantitative data. DLBCL, diffuse large B-cell lymphoma; COO, cell of origin; DE, BCL-2/MYC double expressors; SN, single nodal; SE, single extranodal; GCB, germinal center B-cell; GI, gastrointestinal; LN, lymph node; WR, Waldeyer's ring.

Targeted sequencing, WES, and WGS were performed on 51, 51, and 14 patients, respectively, including 52 of 126 SN patients and 64 of 163 SE patients with available tumor tissue samples. A total of 55 genes related to the tumorigenesis of DLBCL according to literature were analyzed (Figure 2A). At least one mutation was detected in 100/116 (86.2%) patients. The most frequently mutated genes (>10%) included *PIM1* (22/116, 19.0%), *TET2* (20/116, 17.2%), *KMT2D* (17/116, 14.7%), *BTG2* (16/116, 13.8%), *BTG1* (15/116, 12.9%), *MYD88* (15/116, 12.9%), *ARID1A* (13/116, 11.2%), *HIST1H1E* (13/116, 11.2%), *MPEG1* (13/116, 11.2%), *TNFAIP3* (13/116, 11.2%), *TP53* (13/116, 11.2%), *CREBBP* (12/116, 10.3%), *FAS* (12/116, 10.3%), *GNA13* (12/116, 10.3%), and *TMSB4X* (12/116, 10.3%). No significant differences in total mutation frequency between SN and SE groups were observed (Figure 2B). As for individual gene mutation (Supplementary Table 1), significantly increased mutations in *MPEG1* (19.2 vs. 4.7%, *P* = 0.014) were observed in SN than SE patients (Figure 2C). Among common sites of involvement including GI tract, lymph node, Waldeyer's ring, and breast, genetic aberrations of *PIM1, TET2, KMT2D, BTG2, BTG1*, and *MYD88* were assessed (Figure 2D). Patients with lymphoma involvement in the GI tract had significantly decreased *PIM1* (9.1 vs. 25.0%, *P* = 0.034) and *MYD88* (0 vs. 20.8%, *P* = 0.001) mutations than those without GI tract involvement. Patients with lymphoma involvement in breast had higher *MYD88* (36.4 vs. 10.5%, *P* = 0.049) mutations than those without breast involvement.

#### Gene Expression Pattern

**Figure 3:**
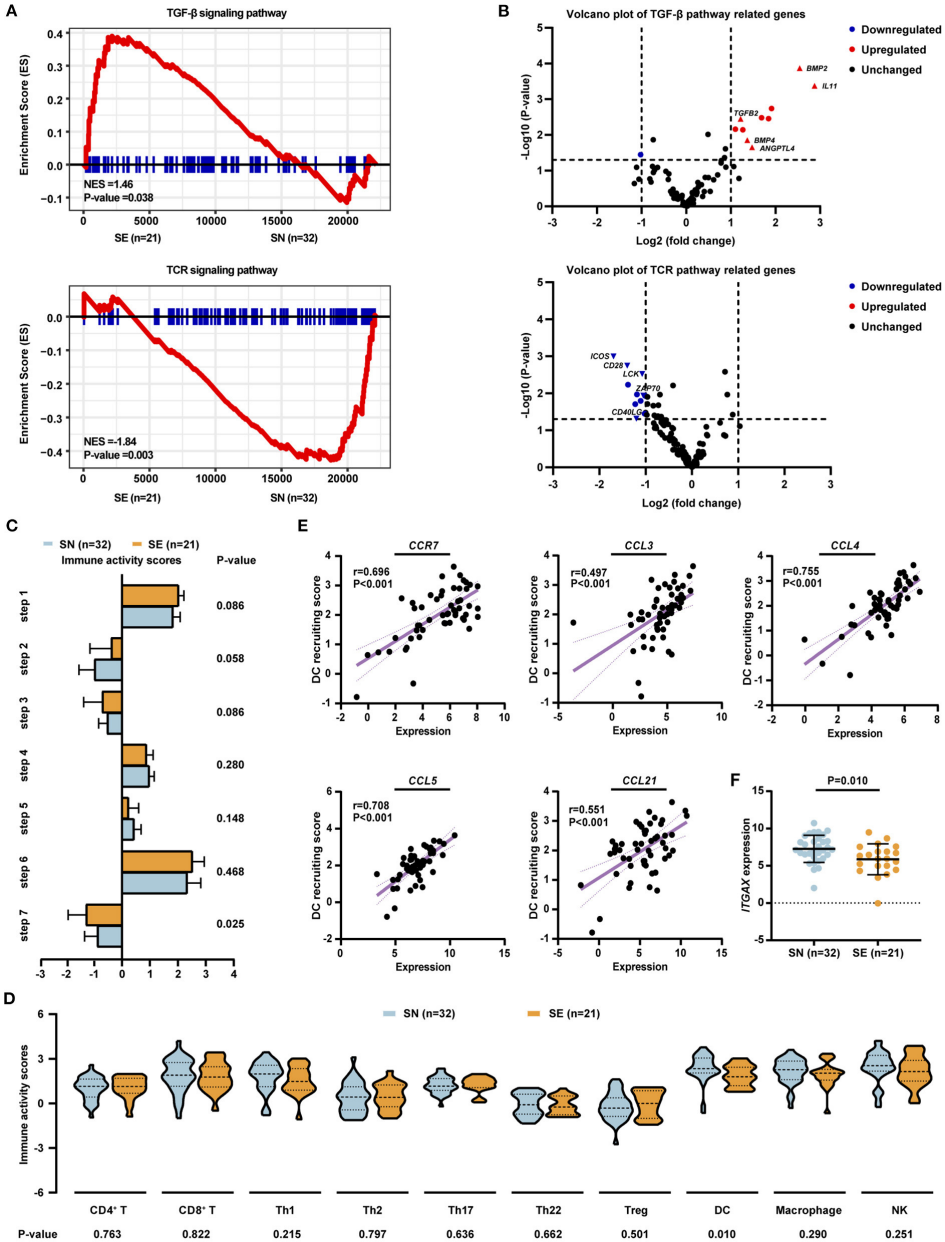
Gene expression pattern and tumor microenvironment profile of patients with localized DLBCL (*n* = 53). **(A)** Significantly altered signaling pathways identified by RNA-seq, including 32 patients with SN and 21 patients with SE involvement. Enrichment plots of TGF-β signaling pathway (upper panel) and TCR signaling pathway (lower panel) by GSEA analysis. **(B)** Volcano plots of gene expression patterns involved in TGF-β signaling pathway (upper panel) and TCR signaling pathway (lower panel). **(C)** Anti-cancer immunity activity scores for seven steps of the cancer–immunity cycle in SN and SE patients. **(D)** Immunity activity scores for recruiting immune cells in SN and SE patients. **(E)** Correlations between dendritic cell recruiting activity and the expression level of chemokines and chemokine receptors including *CCR7, CCL3, CCL4, CCL5*, and *CCL21* as analyzed by Spearman's correlation-test. **(F)** Expression level of dendritic cell marker *ITGAX* in SN and SE patients. Error bars represent lower and upper quartiles. *P*-values were calculated using independent-sample *t*-test or Mann–Whitney *U*-test. DLBCL, diffuse large B-cell lymphoma; SE, single extranodal; SN, single nodal; Treg, regulatory T cell; DC, dendritic cell; NK, natural killer cell.

RNA sequencing was performed on 32 of 126 SN patients and 21 of 163 SE patients. The SN and SE patients differed significantly in gene expression pattern, with 1,894 genes differentially expressed (Supplementary Table 2). Of those, 790 genes were upregulated in the SN group, while 1,104 genes were upregulated in the SE group. Compared with SN patients, SE patients were associated with upregulation of the transforming growth factor-beta (TGF-β) signaling pathway and downregulation of T-cell receptor (TCR) signaling pathway (Figure 3A). Among genes related to the TGF-β signaling pathway, the expression level of *TGFB2, BMP2*, and *BMP4* was significantly increased in SE than SN patients (Figure 3B). Downstream molecules of the TGF-β signaling pathway related to tumor metastasis, including *ANGPTL4* and *IL11*, were also significantly upregulated in SE than SN patients (Figure 3B). As for genes associated with the TCR signaling pathway, the expression level of *ZAP70, LCK, CD40LG, CD28*, and *ICOS* was significantly decreased in SE than SN patients (Figure 3B).

#### Tumor Microenvironmental Pattern

Tumor microenvironment was evaluated by a web server TIP using RNA sequencing data (20). Anti-tumor immune response is generated through a series of stepwise events which are referred to the cancer–immunity cycle, including release of cancer cell antigens (step 1), cancer antigen presentation (step 2), priming and activation (step 3), trafficking of immune cells to tumors (step 4), infiltration of immune cells into tumors (step 5), recognition of cancer cells by immune cells (step 6), and killing of cancer cells (step 7) (21). Among these seven steps, significantly lower immune activity scores of killing of cancer cells (−1.329 vs. −0.905, *P* = 0.025) were observed in SE, as compared to SN patients, while the other six steps showed no obvious differences between SN and SE groups (Figure 3C). As for specific immune cells, SE exhibited a significantly lower recruiting activity of dendritic cells (1.644 vs. 2.199, *P* = 0.010) than SN patients (Figure 3D). Interactions between dendritic cells and chemokines as well as chemokine receptors of the tumor microenvironment were evaluated. The expression level of *CCR7, CCL3, CCL4, CCL5*, and *CCL21* was positively correlated with the recruiting activity of dendritic cells (Figure 3E). In addition, the expression of dendritic cell marker *ITGAX* (5.862 vs. 7.261, *P* = 0.010) was significantly decreased in SE as compared to SN patients (Figure 3F).

### Survival Analysis

**Figure 4:**
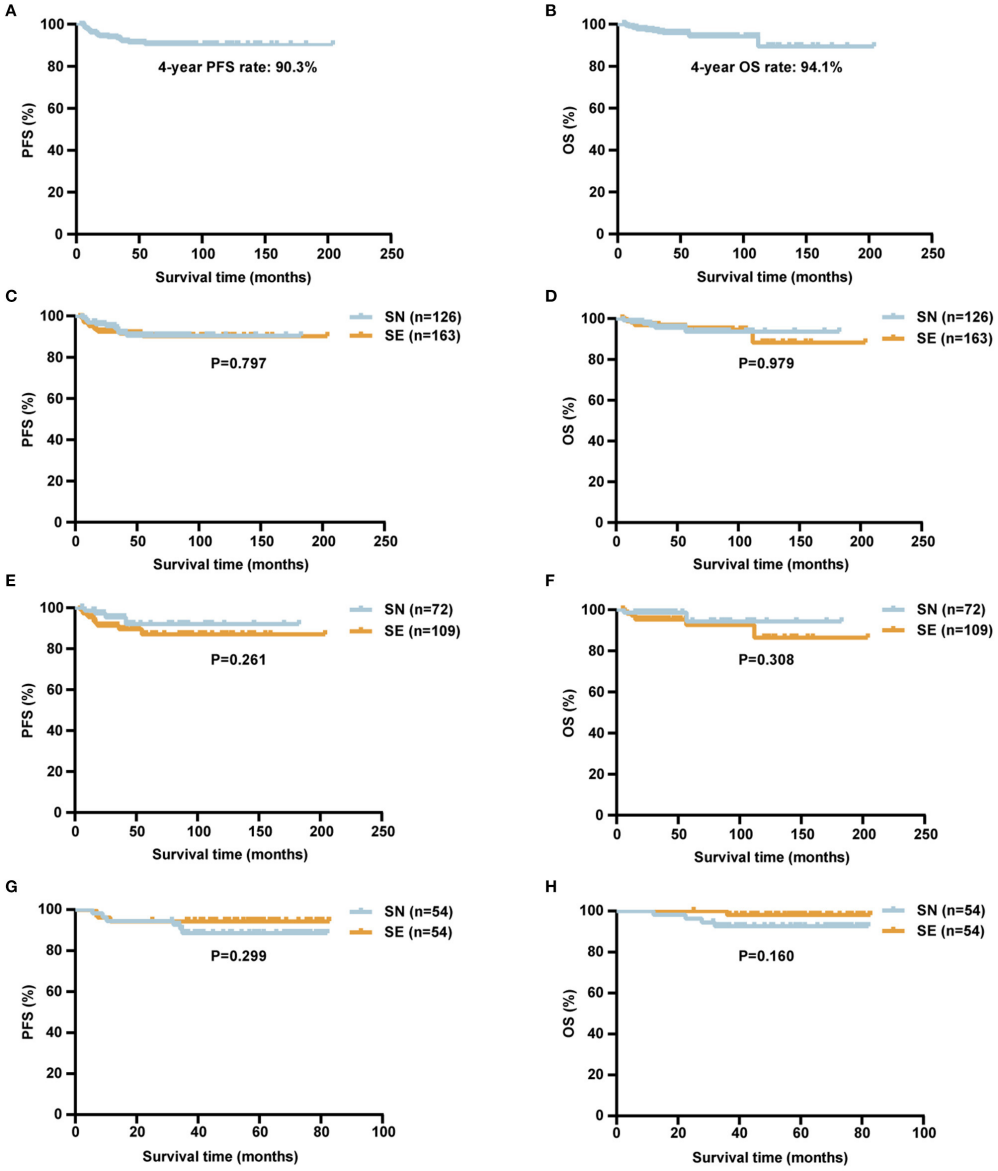
Survival curves of patients with localized DLBCL (*n* = 289). **(A,B)** Kaplan–Meier survival curves of PFS **(A)** and OS **(B)** of localized DLBCL patients. **(C,D)** Kaplan–Meier survival curves comparing PFS **(C)** and OS **(D)** of localized DLBCL patients according to SN (*n* = 126) or SE (*n* = 163) involvement. **(E,F)** Kaplan–Meier survival curves comparing PFS **(E)** and OS **(F)** of localized DLBCL patients according to SN (*n* = 72) or SE (*n* = 109) involvement in the retrospective cohort. **(G,H)** Kaplan–Meier survival curves comparing PFS **(G)** and OS **(H)** of localized DLBCL patients according to SN (*n* = 54) or SE (*n* = 54) involvement in the NHL-001 cohort. *P*-values were calculated using log-rank test. DLBCL, diffuse large B-cell lymphoma; PFS, progression-free survival; OS, overall survival; SN, single nodal; SE, single extranodal.

The median follow-up time was 49.5 (5.1–203.9) months. For a total of 289 stage I DLBCL patients, the 4-year PFS and OS rates were 90.3 and 94.1%, respectively (Figures 4A,B). Among all 289 patients, the 4-year PFS and OS rates were 90.6 and 93.7% in SN group and 90.2 and 94.4% in SE group, respectively (Figures 4C,D). In 181 patients of the retrospective cohort, the 4-year PFS and OS rates were 91.9 and 94.4% in SN group and 87.2 and 92.5% in SE group, respectively (Figures 4E,F). In 108 patients of the prospective cohort, the 4-year PFS and OS rates were 88.5 and 92.4% in SN group and 94.4 and 97.9% in SE group, respectively (Figures 4G,H).

In univariate analysis, serum LDH >3 upper limit of normal (ULN) was significantly prognostic for inferior PFS and OS (Table 3). Other clinical or pathological factors including age, ECOG performance status, specific extranodal sites, Hans, and BCL2/MYC double expressors had no obvious influence on either PFS or OS. In addition, common sites of lymphoma involvement including GI tract, lymph node, Waldeyer's ring, and breast had no significant impact on PFS or OS. Among oncogenic mutations, *ATM* mutations were prognostic for inferior PFS. In multivariate analysis, serum LDH >3 ULN was an independent adverse prognostic factor for OS (Supplementary Table 3). The 4-year OS rate was 60.0% for patients with serum LDH >3 ULN, significantly shorter than those with serum LDH≤3 ULN (94.7%, *P* < 0.001).

**Table 3:** Univariate analysis of predictors for PFS and OS in patients with localized DLBCL (*n* = 289).

**Variable**	**PFS**		**OS**	
	**HR (95% CI)**	***P*-value**	**HR (95% CI)**	***P*-value**
**Age**				
>60 vs. ≤60 years	2.022 (0.908–4.502)	0.085	2.606 (0.903–7.519)	0.076
**Performance status**				
≥2 vs. 0–1	1.345 (0.182–9.966)	0.772	2.467 (0.319–19.049)	0.387
**Serum LDH**				
>1 to ≤3 ULN vs. ≤ULN	1.205 (0.408–3.560)	0.736	0.472 (0.061–3.665)	0.473
>3 ULN vs. ≤ULN	6.158 (1.426–26.591)	0.015	8.395 (1.824–38.650)	0.006
**Specific extranodal sites** ^**a**^				
Yes vs. no	0.870 (0.372–2.033)	0.747	1.655 (0.578–4.739)	0.348
**GI tract involvement**				
Yes vs. no	0.888 (0.380–2.074)	0.783	1.685 (0.588–4.827)	0.332
**Lymph nodal involvement**				
Yes vs. no	0.965 (0.413–2.255)	0.934	0.540 (0.150–1.937)	0.344
**Waldeyer's ring involvement**				
Yes vs. no	0.871 (0.205–3.711)	0.852	3.056 (0.836–11.171)	0.091
**Breast involvement**				
Yes vs. no	0.994 (0.234–4.229)	0.994	0.044 (0.000–192.361)	0.464
**Hans classification (** ***n*** **=** **256)**				
GCB vs. non-GCB	1.293 (0.561–2.984)	0.546	2.571 (0.774–8.541)	0.123
**DE (** ***n*** **=** **249)**				
Positive vs. negative	1.974 (0.717–5.436)	0.188	1.950 (0.527–7.213)	0.317
***ATM*** **mutations (** ***n*** **=** **116)** ^**b**^				
Positive vs. negative	4.317 (1.143–16.305)	0.031	NA	NA

a *Extranodal sites of gastrointestinal tract, liver, and lung according to NCCN-IPI*.

b *Univariate analysis of gene mutations for OS was not obtained for only three deaths occurred in 116 patients*.

*PFS, progression-free survival; OS, overall survival; DLBCL, diffuse large B-cell lymphoma; HR, hazard ratio; 95% CI, 95% confidence interval; LDH, lactate dehydrogenase; ULN, upper limit of normal; GI tract, gastrointestinal tract; GCB, germinal center B-cell; DE, BCL2/MYC double expressors*.

## Discussion

Among localized DLBCL patients, 56.4% were extranodal in origin, consistent with the previous report (22). The GI tract was the most common site of extranodal involvement. Clinically, the majority of localized DLBCL patients presented young age, good ECOG performance status, normal LDH, and no bulky tumors. Significantly increased percentage of low-intermediate risk NCCN-IPI was observed in SE patients due to extranodal involvement of the GI tract, liver, and lung (5). Pathologically, 44.9% of localized DLBCL patients were considered as GCB subtype, similar to the ratio of 42% in total DLBCL (6). Patients with BCL2/MYC double expressors accounted for 14.5% of localized DLBCL patients, while this ratio is up to 20–30% in total DLBCL (23). Meanwhile, SN and SE patients exhibited similar patterns of distribution regarding COO subtype and BCL2/MYC double expressors.

In the rituximab era, the treatment outcome of stage I DLBCL patients was satisfactory. Our study observed that, among 289 patients, the 4-year PFS and OS rates were 90.3 and 94.1%, respectively, much higher than those in patients receiving chemotherapy alone (24). Besides that, extranodal involvement showed no obvious influence on either PFS or OS in localized DLBCL, which seems contradictory with a previous report that addressed the inferior survival of extranodal disease (9). This may be attributed to the different study enrollments between two studies. Moreover, compared with the previous study (9), our study included more SE patients with GI tract involvement that was related to favorable outcomes but less SE patients with bone involvement that was related to unfavorable outcomes (17). As reported in DLBCL (25), serum LDH was also recognized as an unfavorable prognostic factor in localized DLBCL, indicating that more potentially effective immunochemotherapy regimen should be applied in this subset of localized DLBCL patients to improve their outcome. Among oncogenic mutations, *ATM* mutations were related to inferior PFS. As an important cell cycle checkpoint kinase, *ATM* mutations also predicted inferior prognosis in GCB–DLBCL patients (26). However, *TP53* mutations did not have any effect on clinical prognosis, probably due to the limited number of *TP53*-mutant patients and different mutation types in our study. Therefore, multicenter clinical cooperation should be carried out using a matched patient cohort with similar distribution of specific extranodal sites.

As for the molecular features, most frequently altered genes in localized DLBCL included *PIM1, TET2, KMT2D, BTG2, BTG1, MYD88, ARID1A, HIST1H1E, MPEG1, TNFAIP3, TP53, CREBBP, FAS, GNA13*, and *TMSB4X*, which were also reported to be commonly mutated in DLBCL (27, 28). Of note is that increased *MPEG1* mutations were shown in SN than SE patients. *MPEG1* encodes a pore-forming protein, Perforin-2, which is crucial for anti-bacterial defense in human cells (29). With a high mutation rate in DLBCL, the functions of *MPEG1* mutations need to be investigated further. In concordance with previous reports in DLBCL (11, 13, 30), *MYD88* and *PIM1* mutations were frequent in localized DLBCL patients with breast involvement while rare in those with GI tract involvement. As for oncogenic cascades, the TGF-β signaling pathway has been reported to be associated with extranodal involvement in DLBCL (31, 32). Indeed key members of the TGF-β superfamily including TGFB2, BMP2, and BMP4, as well as functional molecules of the TGF-β signaling pathway including ANGPTL4 and IL11 (31), were significantly increased in SE patients. Recently, anti-TGF-β therapies have demonstrated potent anti-tumor activity in several clinical studies (33). Therefore, therapeutic targeting of the TGF-β signaling pathway may be effective in counteracting the extranodal involvement in DLBCL. Meanwhile, the TCR signaling pathway was downregulated in SE patients. Here proximal TCR signaling molecules ZAP70 and LCK (34) and costimulatory molecules CD40LG, CD28, and ICOS (35, 36) were also significantly decreased in SE patients. Besides that, evaluation of the tumor microenvironment by the cancer–immunity cycle revealed that, compared with SN patients, SE patients exhibited a lower activity of killing of cancer cells and recruiting dendritic cells. Dendritic cells are antigen-presenting cells and crucial in T-cell priming and antitumor activity (37). Chemokines and chemokine receptors associated with recruiting and homing of dendritic cells including CCR7, CCL21, CCL3, CCL4, and CCL5 (38–40) showed a positive correlation with the recruiting activity of dendritic cells. Moreover, the dendritic cell marker ITGAX, which was related to superior survival in DLBCL patients (41), was significantly decreased in SE patients. Therefore, localized DLBCL with extranodal involvement could be featured with a relatively immunosuppressive tumor microenvironment, indicating some immunomodulatory agents as the potential effective alternatives for targeting extranodal lesion.

In conclusion, localized DLBCL patients may differ from nodal to extranodal involvement and present distinct genetic alterations, gene expression pattern, and tumor microenvironment profile, which could provide a clinical rationale for future mechanism-based therapy in localized DLBCL.

## Data Availability Statement

The datasets presented in this study can be found in online repositories. The names of the repository/repositories and accession number(s) can be found below: https://www.biosino.org/node, OEP001143.

## Ethics Statement

The studies involving human participants were reviewed and approved by Ruijin Hospital Ethics Committee. The patients/participants provided their written informed consent to participate in this study.

## Author Contributions

WZ, PX, LW, and SC designed the study. WQ, HY, LD, QSo, XJ, and ZL acquired data. WQ, QSh, WZ, PX, DF, HH, and JH analyzed the data and made the figures. WZ, PX, WQ, and DF drafted the manuscript. All authors contributed to the article and approved the submitted version.

## Conflict of Interest

The authors declare that the research was conducted in the absence of any commercial or financial relationships that could be construed as a potential conflict of interest.

## Publisher's Note

All claims expressed in this article are solely those of the authors and do not necessarily represent those of their affiliated organizations, or those of the publisher, the editors and the reviewers. Any product that may be evaluated in this article, or claim that may be made by its manufacturer, is not guaranteed or endorsed by the publisher.
